# Is there a Lyme-like disease in Australia? Summary of the findings to date

**DOI:** 10.1016/j.onehlt.2016.03.003

**Published:** 2016-04-07

**Authors:** Melissa Judith Chalada, John Stenos, Richard Stewart Bradbury

**Affiliations:** a School of Medical & Applied Sciences, Central Queensland University, Rockhampton, Queensland, Australia; b Australian Rickettsial Reference Laboratory, Barwon Health, Geelong, Victoria, Australia

**Keywords:** Lyme-like, Lyme, Borreliosis, Tick-borne, Australia

## Abstract

Lyme Borreliosis is a common tick-borne disease of the northern hemisphere caused by the spirochaetes of the *Borrelia burgdorferi* sensu lato (*B. burgdorferi* s. l.) complex. It results in multi-organ disease with arthritic, cardiac, neurological and dermatological manifestations. In the last twenty-five years there have been over 500 reports of an Australian Lyme-like syndrome in the scientific literature. However, the diagnoses of Lyme Borreliosis made in these cases have been primarily by clinical presentation and laboratory results of tentative reliability and the true cause of these illnesses remains unknown. A number of animals have been introduced to Australia that may act as *B. burgdorferi* s. l. reservoirs in Lyme-endemic countries, and there are some Australian *Ixodes* spp. and *Haemaphysalis* spp. ticks whose geographical distribution matches that of the Australian Lyme-like cases. Four published studies have searched for *Borrelia* in Australian ticks, with contradicting results. The cause of the potential Lyme-like disease in Australia remains to be defined. The evidence to date as to whether these illnesses are caused by a *Borrelia* species, another tick borne pathogen or are due to a novel or unrelated aetiology is summarised in this review.

## 1 Introduction

Lyme Borreliosis is a common tick-borne disease of the northern hemisphere. It is caused by spirochaetes of the *Borrelia burgdorferi* sensu lato (*B. burgdorferi* s. l.) complex. Typically, the disease first presents with an erythema migrans rash at the site of the tick bite, followed by flu-like symptoms and later by debilitating arthritic, dermatological and neurological manifestations. The bacteria are transmitted by *Ixodes* species ticks, although other Ixodidae ticks [1], [2], [3], [4] and haematophagous arthropods [2], [4], [5], [6], [7], [8], [9], [10], [11], [12], [13], [14] have been implicated in carrying the bacteria. Bacterial reservoirs of the disease are usually small mammals, birds and occasionally reptiles [15], [16]. The presence or absence of Lyme disease (or a Lyme-like disease) in Australia remains a contentious issue with varying opinions being held by medical practitioners, scientists and lay stake holders while the aetiological agent remains undetermined.

In response to the continued controversy and media attention regarding the possibility of there being Lyme Borreliosis in Australia, the Australian Government Chief Medical Officer, Professor Chris Baggoley, established the Clinical Advisory Committee on Lyme Disease (CACLD) in 2013 [17]. The purpose of this committee was to advise the Chief Medical Officer on the following points:

1. The extent to which there is evidence of *Borrelia* species causing illness in humans in Australia
2. The most appropriate laboratory diagnostic testing algorithms (best world practice) for persons who have suspected Borreliosis in Australia
3. The most appropriate treatments for Borreliosis in Australia
4. The most appropriate ways to disseminate information to health professionals and the general public on Borreliosis/Lyme disease
5. The requirements for further research into Borreliosis in Australia, and the generation of appropriate new questions relevant to the terms of reference.

Furthermore, the Australian Government Department of Health commissioned a scoping study [18] to identify the gaps in scientific evidence surrounding the causative agent of the Australian Lyme-like disease. Subsequently, upon advice from the CACLD, the Australian public was called upon to review and contribute to the scoping study, and 36 submissions were obtained in total. All points raised were considered individually and then collated, culminating in the following twelve considerations [18]:

1. Does *B. burgdorferi* s. l. occur in Australian ticks, and especially in *Ixodes holocyclus*?
2. Do other Australian tick species transmit Lyme Borreliosis?
3. Can Australian ticks be infected with, maintain, and transmit *B. burgdorderi* s. l.?
4. Can we find better diagnostic tools to search for Lyme Borreliosis?
5. Is there an indigenous species of *Borrelia* in Australia able to infect humans and able to cause a Lyme disease-like syndrome?
6. Do other possible pathogens occurring in Australian ticks cause a Lyme disease-like syndrome?
7. Are there any relapsing fever group *Borrelia* species in Australia?
8. Can *B. burgdorferi* s. l. be detected with any certainty in erythema migrans rashes following a tick bite, as demonstrated by PCR and/or culture of biopsy specimens?
9. Is there an immune response to *B. burgdorferi* s. l. or to any other possible agent in the sera of patients presenting with a Lyme disease-like syndrome?
10. Are there any *B. burgdorferi*-specific IgG antibodies in the sera of patients with Lyme disease-like syndrome?
11. If there is evidence found to indicate the presence of Lyme Borreliosis or a Lyme disease-like syndrome in Australia, what is the geographic spread of cases?
12. Are there other potential vectors that could transmit *Borrelia* in Australia?

Further to the above identified knowledge gaps, during the course of this literature review, the authors will consider two further points of investigation:

1. Could native Australian animals act as reservoirs of *B. burgdorferi* s. l.?
2. Could introduced animals such as foxes, hares, placental mice and rats act as reservoirs of *B. burgdorferi* s. l. in Australia?

The purpose of this review is to assess the current situation of the controversial Lyme or Lyme-like illness reported by some to be present in Australia. The existing evidence is explored and areas require further investigation are identified. Alternative infectious and non-infectious diagnoses are also considered.

## 2 Potential reservoirs of Lyme Borreliosis-causing *Borrelia* species in Australia

If a *Borrelia* causing a Lyme-like disease is present in Australia, importation or native evolution are both possible origins of the causative agent. Such an agent might be a known *Borrelia* species or a novel, as yet undescribed microbial pathogen.

### 2.1 *Borrelia* in introduced animals

In the 1900s, two species of *Borrelia* were introduced to Australia via the agricultural industry. These were *Borrelia theileri*, the worldwide cause of bovine Borreliosis [19], and *Borrelia anserina*, the worldwide agent of avian spirochaetosis [20]. *B. theileri* has been reported in cattle of Queensland and New South Wales [21], [22], [23] and *B. anserina* has infected poultry of Victoria and the Northern Territory [23], [24], [25]. *B. theileri* is transmitted in Australia by the cattle tick *Rhipicephalus* (*Boophilus*) *australis* [21], [26] while the vector of *B. anserina* is *Argas persicus* s. l. [27]*. Argas persicus* ticks have been observed in all states of Australia except for Tasmania, and *R. australis* is distributed along the northern and eastern coasts of Australia [26]. *R. australis* may occasionally bite humans [26]. Neither *B. anserina* nor *B. theileri* belong to the *B. burgdorferi* s. l. complex, nor have they ever been described as causing a Lyme-like illness in humans.

If Lyme Borreliosis was present in Australia, it is reasonable to expect that its presence would be prominent in livestock, domestic animals and particularly feral deer, as is the case with Lyme Borreliosis in the northern hemisphere. However, very few cases of a Lyme-like illness in Australian animals are present in the veterinary literature. Lyme Borreliosis was reported in two cows at Camden, New South Wales in 1989 [28]. These cows were previously infested with *Haemaphysalis longicornis* (see Section 2.5) and presented with fever, anaemia, poor condition and polyarthritis. The diagnosis of Lyme Borreliosis was made in the first cow on the presence of spirochaetes in the synovial stroma and the second by positive IFA Lyme serology. However, from the images of spirochaetes from the first case described in the paper it is unclear if these represent true spirochaetes or artefact. Ephemeral fever, chlamydiosis, *Mycoplasma bovis* and “other septicaemic bacteria” were ruled out in the cows, but it is unspecified if *B. theileri* was one of the septicaemic bacteria considered. *B. theileri* antibodies may cross-react with *B. burgdorferi* s. l. [29]. This agent can cause fever and anaemia, although is not associated with polyarthritis [30], [31], [32], [33]. Conversely, polyarthritis has been associated with *B. burgdorferi* s. l. in animals in the northern hemisphere [30]. However, true Lyme Borreliosis was not confirmed by the diagnostic techniques performed in these cases, and it is possible that these cases were *B. theileri* infection. Overall, the relative absence of reports of veterinary cases of Lyme or a Lyme-like disease in Australia suggests the absence of traditional Lyme Borreliosis causing agents in the country.

### 2.2 *Borrelia* in native animals

Reports of “*Borrelia* species” in Australian native animals appear to be localised to Queensland. In Brisbane, spirochaetes observed in blood films of bandicoots, and in western Queensland spirochaetes were seen in blood films of kangaroos were both identified as a novel *Borrelia* species [34]. The identification of these *Borrelia* to the species level was not determined, and their place in the phylogeny of the *Borrelia* genus remains unknown. Molecular characterisation methods were not available at the time and morphological appearance alone was used to classify these into the *Borreli*a genus. Due to their presence in blood films, it is hypothesized that these spirochaetes were likely to be relapsing fever *Borrelia* rather than Lyme-causing *Borrelia*, since the latter generally have a much lower spirochaetal load in the bloodstream than the former. Once again, modern phylogenetic analysis techniques that would have definitively placed these spirochaetes into Lyme-causing or relapsing fever *Borrelia*, or another genera of spirochaete altogether were not available to confirm these diagnoses. In 1956, Pope and Carley isolated a spirochaetes from one native rat (*Rattus villosissmus*) out of twenty-seven dead and dying rats tested in Richmond, north-western Queensland [35] and named it *Borrelia queenslandica* [36]. Attempts to infect a human volunteer with this spirochaetes were unsuccessful [37] and attempts to transmit the spirochaete from mouse to mouse via the Argasid tick *Ornithodorus gurneyi* were also unsuccessful [38]. Due to loss of all isolates, whether *B. queenslandica* is a part of the *B. burgdorferi* s. l., a relapsing fever group or another genus of spirochaete cannot now be determined. However, the lack of pathogenicity in the human volunteer are counterindicative of this organism being the causative agent of the Australian Lyme-like disease considered in this paper.

### 2.3 Spread of *Borrelia* by migratory birds

Birds play an important role in the perpetuation of ticks and *B. burgdorferi* s. l. in North America [39], [40], Europe and Asia [41], [42], [43]. More significantly there is evidence that bird migration results in a wider dispersion of Lyme-causing *Borrelia*. Examples of this include the detection of *B. burgdorferi* s. l. in migratory songbirds across Canada [40] and the transport of *Borrelia garinii* via birds migrating from mainland Asia to Japan [42]. Most relevant to the Australian situation is the worldwide dispersal of seabird species and the seabird tick *Ixodes uriae* [44], [45], [46]. *B. garinii*, a species known to cause Lyme Borreliosis, has been detected in *I. uriae* not just in the northern hemisphere [46], [47], but also in southern hemisphere locations including Campbell Island off New Zealand, the Crozet Islands in the southern Indian Ocean and the Falkland Islands off South America [46]. This transhemispheric dispersal of *B. garinii* may be not just due to the spread of infected ticks, but also by seabirds acting as *B. garinii* reservoirs. However, the theoretical spread of *B. garinii* from seabirds to humans and even other birds and mammals, is unlikely, as generally the seabirds and their ticks are restricted to the open sea, remote islands, and peninsulas where contact with other animals is rare [46]. The ticks of seabirds along the Australian coast have not to date been investigated for *Borrelia*.

### 2.4 Introduced animals identified as Lyme reservoirs overseas

A number of non-native mammals have been introduced to mainland Australia since its settlement [48], some of which are known reservoirs of *B. burgdorferi* s. l. in the northern hemisphere.

Several introduced animals found in Australia, including the black rat (*Rattus rattus*), the house mouse (*Mus musculus*), the brown hare (*Lepus europaeus*), several species of deer and to lesser extents the red fox (*Vulpes vulpes*) and the Norwegian rat (*Rattus norvegicus*) are known to be reservoirs of *B. burgdorferi* s. l. in the northern hemisphere. Most of these animals have established widespread populations in Australia since their introduction, excepting the Norwegian rat, which has established a localised population only [48]. In Australia, *R. rattus and R. norvegicus* in Australia are parasitised by *I. holocyclus* and *Ixodes tasmani* ticks [26], [49], *M. musculus* is parasitized by *I. tasmani*, and *L. europaeus* is parasitized by *H. longicornis* [26]*.* No studies on the ticks commonly parasitising *L. europaeus* in Australia have been performed, but these hares tend to occur in open grassland, which is not a preferred habitat of *Ixodes* ticks. To date, no investigations have been conducted into the presence or absence of *B. burgdorferi* s. l. in introduced undomesticated animals of Australia.

### 2.5 Likely tick vectors of *B. burgdorferi* s. l. in Australia

Overall very little evidence exists of the transmission of a potential Lyme-like disease by Australian ticks. It is hypothesised that if ticks are transmitting *B. burgdorferi* s. l. in Australia, the tick species would parasitize a number of hosts including humans, and would likely (but not necessarily) be of the *Ixodes* genus, as this is the genus that transmits Lyme Borreliosis in the northern hemisphere. The following information is intended only to identify the need for further research in testing for the presence of *B. burgdorferi* s. l. in wild populations of these ticks, and if *B. burgdorferi* s. l. is present, their transmission competency.

In the northern hemisphere, the Lyme-causing *Borreliae* are transmitted mainly by *Ixodes* species ticks. Nineteen species of *Ixodes* have been described in Australia [49], many of which have only a small geographical distribution (e.g. *Ixodes vestitus* and *Ixodes myrmecobii* are localised to Western Australia) or a limited host range (e.g. *Ixodes vespertillionis* is confined to bats and *Ixodes ornithorhynchi* to the platypus) [49]. It should be noted that the main ticks that transmit Lyme Borreliosis in the northern hemisphere are the black-legged ticks (the *ricinus* complex [50]) and that there are none of this group found in Australia. *I. holocyclus* and *I. tasmani* appear to have the widest geographical spread of the Australian *Ixodes* species while also having a large range of potential hosts. Furthermore, *I. myrmecobii* occurs in WA and belongs to the same subgenus (*Sternalixodes*) as *I. holocyclus* [51].

*I. tasmani* is the most abundant species of tick in Tasmania but is also found throughout Victoria, along the coastal and sub coastal areas in New South Wales and Queensland and in parts of southeast South Australia and southwest Western Australia [26]. *I. tasmani* has a broad range of hosts, but rarely bite humans, making it a candidate as a tick-borne disease reservoir and bridge vector of any putative tick borne Lyme-like agent in Australia, but an unlikely candidate vector to humans. Examples of hosts it parasitises include possums, bandicoots, wallabies, native rats, introduced rats, dogs, cats, horses and humans. No work has been published regarding the potential vector competence of *I. tasmani* for *B. burgdorferi* s. l.

**Fig. 1:**
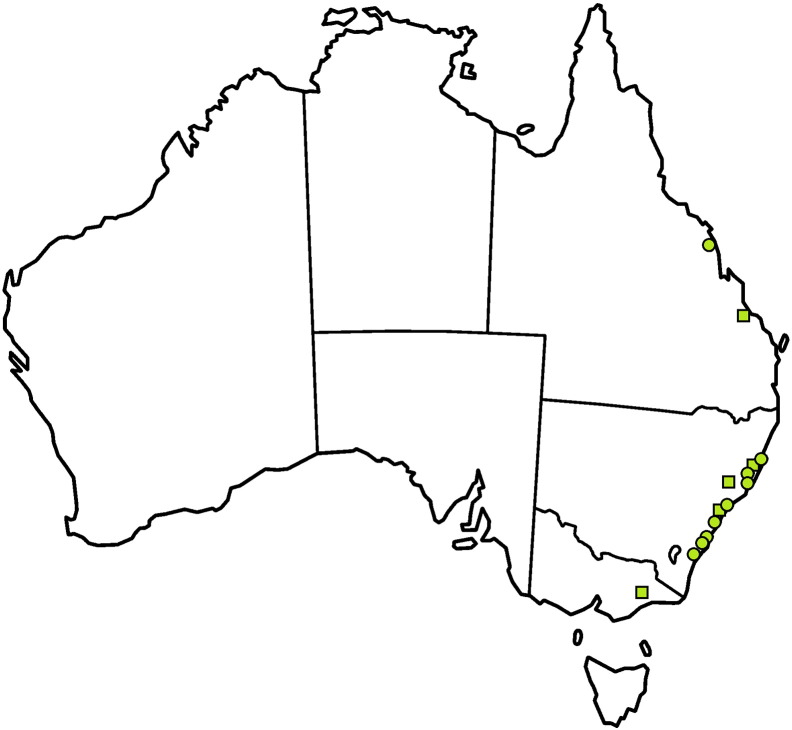
Locations of Australian Lyme-like cases published in the scientific literature. Specific location based on town, suburb or GPS coordinates. Approximate location based on broad location description, e.g. “rural Victoria” or “Hunter Valley”.

*I. holocyclus*, colloquially known as the “paralysis tick”, has an extensive host range including, but not limited to, domestic animals such as cats, dogs, chickens and other fowl, ducks and man [26]. Native animal hosts include wallabies, kangaroos, bandicoots, possums and dingoes [26]. *I. holocyclus* is distributed along coastal areas of northern and eastern coasts of Queensland and New South Wales, Victoria and Tasmania. In southern Queensland and northern New South Wales its range also extends somewhat further inland [49]. This geographic distribution coincides with that of the Lyme-like disease cases reported in the scientific literature (Fig. 1). Although there are anecdotal reports of a Lyme-like illness being present in Western Australia, outside of the range of *I. holocyclus*, no cases have been published in the scientific literature. However, in a vector competence experiment, *I. holocyclus* was able to ingest but not transmit the JD1 strain of *B. burgdorferi* s. s. [52]. Whilst this finding does not preclude the capacity of *I. holocyclus* to transmit other *B. burgdorferi* s. l. species or strains, it does infer a likelihood of poor vector competence for this species.

In the northern hemisphere, *B. burgdorferi* s. l. has also been detected in Ixodidae (hard tick) of genera other than *Ixodes* [1], [2], [3], [4] and even in haematophagous arthropods including lice [2], fleas [4], [5], keds [2], [6], mites [7], [8], [9], flies [10], [11], [12] and mosquitoes [9], [13], [14]. While the transmission capability of these arthropods remains undetermined, it does raise the possibility of Lyme transmission by arthropods other than *Ixodes.* In Australia, other genera of hard ticks include *Amblyomma*, *Haemophysalis*, *Bothriocroton* and *Rhipicephalus.* These vary in their distribution and host range depending on the species. The “bush tick” *H. longicornis* is a native of the south-east coast of Russia, North and South Korea, Japan and China [26] and is believed to have been introduced to Australia in the nineteenth century from Japan [63]. In Australia, *H. longicornis* occupies a large coastline area spanning from central Queensland to south-eastern Victoria but is found especially in Kempsey, New South Wales [26]. A very small area in the south-western corner of Western Australia also contains *H. longicornis* [64]. Similar to *I. holocyclus*, *H. longicornis* parasitises a large number of hosts in Australia including but not limited to cattle, sheep, horses, dogs, cats, hares, domestic fowls, Australian magpies and marsupials [26], but only rarely bites humans [26]. The detection of *B. burgdorferi* s. l. in *H. longicornis* ticks of Japan [65] and China [3] supports the possibility that *H. longicornis* could carry Lyme Borreliosis in Australia.

### 2.6 Studies investigating *Borrelia* in Australian ticks

Four studies have been published that investigated the potential for *B. burgdorferi* s. l. in ticks, both employing culture with or without PCR and in the most recent studies, next generation sequencing.

#### 2.6.1 Wills and Barry 1991

Wills and Barry [66] published preliminary results of their investigations into the presence of *Borrelia* in Australian ticks in a letter to the editor of *The Medical Journal of Australia* in 1991. One-hundred and sixty-seven ticks consisting of *I. holocyclus* and *H. longicornis* were collected from the Hunter Valley and Manning River districts of coastal New South Wales and their midguts were cultured in BSK-II media. Within 8 weeks incubation, motile, rigid spirochaete-like objects (SLOs) were observed in 44% of their *I. holocyclus* cultures and 35% of their *H. longicornis* cultures; a total of 70 Australian tick midgut positive cultures out of 167 cultured. The individual number of *I. holocyclus* midguts and *H. longicornis* midguts cultured was not specified. The presumptive spirochaetes were described as “large, coiled motile bacteria with an irregular rotational movement” and were “morphologically indistinguishable” to the reference strain *B. burgdorferi* (B31). At least four of the spirochaetes isolated shared antigenic epitopes with *B. burgdorferi* as demonstrated by ELISA, immunofluorescence and western blotting, suggestive of *Borrelia* species. However, details of the laboratory methods are not published and the organisms recovered were not made available for confirmation by another laboratory, rendering the experiment unable to be replicated. False positives in the ELISA, immunofluorescence and western blotting cannot be ruled out. No PCR or sequencing has been conducted to confirm the identity of the isolates, and positive *Borrelia* cultures from Australian tick samples have not been reproduced to date. Although this investigation was conducted as a part of the author's (Wills') PhD, no follow-up report to these preliminary findings was ever published in the scientific literature.

Alleged L-form variant “rigid” SLOs of *Borrelia* have been described in other papers, from cultured biopsy and synovial fluid samples from Lyme Borreliosis patients [67], [68], in animal skin sample cultures [69] and in control *Borrelia* cultures subjected to antispirochaetal agents [70], [71]. However, the SLOs in contaminated cultures observed under electron microscopy have been identified by some researchers [69], [72], [73] as large flagella aggregates from the contaminating bacteria, and therefore not indicative of the presence of *Borrelia* spirochaetes. Furthermore, cultures of *Bacillus* have been identified as capable of producing these structures [74]. It is possible that this is what was observed in the cultures conducted by Wills and Barry [66]. However, this does not explain the return of such rigid SLOs to normal, motile spirochaetes after multiple subcultures [66], [67]. SLOs in uncontaminated cultures have been observed by others and can be explained by the flagella passing though filters that block whole bacteria and the flagella then coalesce to form the long SLOs (Doggett, S. pers. comm. 2016). The use of molecular techniques, especially sequencing, would be ideal for confirmation or dismissal of any cultured SLOs as *Borrelia.*

#### 2.6.2 Russell et al. 1994

A comprehensive search for *Borrelia* in Australia conducted by Russell et al. [73] contradicted the findings of Wills and Barry [66]. Approximately 12,000 ticks were collected over three years along the New South Wales coast. Ticks were collected from natural habitats and removed from native and domestic animals, although the animal species are not disclosed. The majority of tick species collected were *I. holocyclus* (7922) followed by *H. longicornis* (2208) and *Haemaphysalis bancrofti* (1092). The remaining 786 ticks consisted of nine other species. Tick midguts were cultured in BSK-II media and screened by dark-field microscopy, although factors including nutritional media components, chemical and physical culture conditions were adjusted in an unspecified number of cultures. Ninety-two cultures of bloodfed ticks revealed SLOs. These SLOs were straight, rigid and uniformly coiled and non-motile and later determined to be bacterial flagella aggregates by electron microscopy. The authors describe “a few” of the 18 SLOs as having tested positive using polyclonal *B. burgdorferi* s. s. antibodies, though none reacted with monoclonal *B. burgdorferi* s. s. antibodies. The study found “no definitive evidence for the existence in Australia of *B. burgdorferi* the causative agent of true Lyme Borreliosis, or for any other tick-borne spirochaete that may be responsible for a local syndrome being reported as Lyme disease”. The authors observed Wills' and Barry's [66] cultured *Borrelia* and found them to be identical to their own SLOs, concluding that Wills' and Barry's cultured SLOs were also contaminant flagella aggregates. Russell et al. also had the advantages of *Borrelia* genus-specific PCR and a much larger sample size over Wills' and Barry's study. The conclusion of Russell et al.'s study – that no spirochaetes were able to be identified through culture or molecular methods in Australian ticks – therefore seems more plausible than the conclusions of Wills and Barry.

#### 2.6.3 Gofton et al. 2015a

A recent study by Gofton et al. found no *B. burgdorferi* s. l. in Australian *I. holocyclus* ticks, but did detect a novel relapsing fever group *Borrelia* [75]. This study tested 109 *I. holocyclus* from around New South Wales, collected over a ten year period. DNA extracted from these ticks was subjected to next generation sequencing to determine the bacteriome of the ticks. Thirty *Ixodes ricinus* ticks collected in Germany were included for comparative purposes. Whilst *B. burgdorferi* s. l. sequences were not recovered from any Australian *I. holocyclus* ticks, nine (30%) of the German *I. ricinus* samples yielded 16SrRNA sequences homologous to either *B. burgdorferi* s. s. or *Borrelia afzelii* [75]. A single Australian *I. holocyclus* taken from an echidna yielded 16SrRNA sequences of an unknown *Borrelia* species, clustering within the relapsing fever group and not the *B. burgdorferi* s. l. group of Borreliae [75].

This work provides further evidence that the cause of the Lyme-like illness in Australia may not be a member of the *B. burgdorferi* s. l. complex. The finding of a novel relapsing fever *Borrelia* in an Australian monotreme does provide evidence for the presence of Borreliae in Australia, but it is not known if this organism can infect humans, and should it do so, it is likely that it would present as a relapsing fever illness rather than with Lyme-like symptoms. These factors limit the likelihood that this novel *Borrelia* species is the cause of the Lyme-like illnesses seen in Australia. The study was limited by the relatively low number of ticks sampled and the limited geographic range from which they were collected. No data was presented regarding the distribution of collection sites (urban, rural or wilderness) within that state.

#### 2.6.4 Gofton et al. 2015b

In the above study, only one species of tick, *I. holocyclus*, was õsampled in this study [76]. Although it is assumed that this is the most likely vector candidate in Australia by many researchers, as noted in Section 2.5 of this review, this species has been shown not to be able to transmit *B. burgdorferi* s. s. in vector competence studies. *H. longicornis*, with its wider geographic range and known competence as a vector of Lyme-causing *Borrelia* in Japan, would be a superior candidate for potential *B. burgdorferi* s. l. transmission in Australia, except that it very rarely bites humans. Further work using the same protocol on a larger cohort of ticks, from an Australia-wide catchment and including other tick species (particularly *H. longicornis*) is warranted. Gofton et al. addressed this requirement in a recently published study of 460 ticks collected from below the line of the tropic of Capricorn in Western Australia and the seaboard Eastern Australia (though one from inland Queensland was included). The ticks were identified as being 279 *I. holocylcus*, 167 *Amblyomma* *triguttatum*, seven *H. bancrofti* and a further seven *H. longicornis*. Midguts of all ticks were subjected to 16s ribosomal RNA PCR and next generation sequencing. A *Borrelia* genus specific *flaB* nested PCR was also performed on all ticks recovered. None of the ticks concerned yielded any *Borrelia* sequences or PCR products [76].

## 3 Relevance of diagnostic techniques to Australia

### 3.1 Diagnosis in the endemic setting

In the Lyme Borreliosis endemic United States of America (USA), serology for Lyme Borreliosis is the diagnostic technique recommended by the Centers for Disease Control and Prevention (CDC) [54]. Serology is conducted by a two tiered approach: firstly, an enzyme-linked immunosorbent assay (ELISA) or immunofluorescence antibody (IFA) test is performed, and if positive, this is followed secondly by an immunoblot. The ELISA or IFA tests may give false-positive reactions in the presence of other infectious, autoimmune or inflammatory conditions [53], [54]. Similarly, not performing the ELISA or IFA step will increase the likelihood of false positives in the immunoblot [57].

The interpretation of the immunoblot depends on the number of bands present. In the USA, where *B. burgdorferi* sensu stricto (*B. burgdorferi* s. s.) is the only causative agent of Lyme Borreliosis, the following criteria are required for diagnosis: An IgM immunoblot is positive if two of the three bands are present: 24 kDa (OspC), 39 kDa (BmpA), and 41 kDa (Fla) [56]. An IgG immunoblot is considered positive if five of the following 10 bands are present: 18 kDa, 21 kDa (OspC), 28 kDa, 30 kDa, 39 kDa (BmpA), 41 kDa (Fla), 45 kDa, 58 kDa (not GroEL), 66 kDa, and 93 kDa [55]. In patients with acute Lyme Borreliosis (less than 30 days) within the USA, the IgM blot has a sensitivity of 58.5% and specificity of 92% to 94% [56]. In patients greater than 30 days after initial infection, the IgG blot has a sensitivity of 83% and specificity of 95% [55].

In Europe and Asia there are a greater number of *B. burgdorferi* s. l. species and strains that cause Lyme Borreliosis than there are in the United States [60], [61]. Different strains of *B. burgdorferi* s. l. may express only some of the antigens detected in immunoblot, may constitutionally lack certain genes for certain proteins, or comprise immunodominant antigens of molecular weights that differ from those typically used in the immunoblot. For these reasons, the immunoblot interpretation using a method developed at one geographic area may not be applicable to other geographic areas. Consequently, standardisation of immunoblotting methods for Lyme Borreliosis diagnosis in Europe and Asia is unfeasible [60], [61]. A number of commercial immunoblot kits and interpretative criteria are available with varying specificity and sensitivity [62].

### 3.2 Confounding factors in serological diagnosis in the non-endemic setting

The CDC diagnostic serological method used for *B. burgdorferi* s. s. is inappropriate for use in the Australian context except for patients with a travel history to endemic countries [59]. It is possible that any theoretical Australian *B. burgdorferi* s. l. species would cause a different serological response in a Lyme Borreliosis patient than the American, Asian or European species. Such antigenic differences could result in false negative serology results. It has been shown that chronic Lyme Borreliosis patients may test seronegative even if they are PCR confirmed or culture confirmed to be infected by *B. burgdorferi* s. l. [58], [77]. This does not necessarily mean that these patients lack an antibody response, but rather the banding pattern in an immunoblot is merely different to that of the standard diagnostic criteria [78]. This must be considered in regard to almost all of the purported Lyme-like illness cases seen in Australia, which almost exclusively [79], [80], [81], [82] present with clinical symptoms correlating to the late (greater than 30 days duration) stage of Lyme Borreliosis.

It is important to consider that in areas not endemic for Lyme Borreliosis, the positive predictive value of the serology test will be low [59]. In endemic areas, patients with other illness and even healthy donors may display at least 5 of the 10 bands required for a positive anti-*B. burgdorferi* IgG western blot result [56]. Furthermore, in the non-endemic setting of Papua New Guinea, 50% of 84 individuals screened for Lyme Borreliosis fitted the CDC serological criteria for Lyme Borreliosis [83]. Further testing of these samples for antibodies to *Treponema pallidum* by microhaemagglutination assay, rapid plasma reagin test, fluorescent treponemal antibody-absorption test, and Western blot all yielded negative results. The pattern of IgG bands seen differed from controls with confirmed Lyme Borreliosis and none of the patient sera inhibited the growth of *B. burgdorferi in vitro*, whilst 69% of Lyme patient sera will do so [83]. It was thought that the false positive Lyme serology results were the consequence of high levels of immunoglobulin or cross-reactive antibodies residents of tropical regions [83]. It is possible this same phenomenon may occur in Australia. While the causative agent of the putative Lyme-like disease remains unknown, any positive or negative Lyme serology results are unreliable.

### 3.3 The RCPA protocol for the diagnosis of Lyme Borreliosis in Australian patients

The many confounding factors influencing Lyme Borreliosis diagnosis in Australia led to the release in 2014 by the Royal College of Pathologists of Australasia (RCPA) of a position statement on the diagnostic laboratory testing for Lyme Borreliosis [59]. This position statement sought to address misinformation regarding the Lyme Borreliosis in Australia and to provide guidance to clinicians in regard to ordering tests for the diagnosis of potential Lyme Borreliosis cases. This very balanced statement noted that Australia was amongst several countries in which the presence of local Lyme Borreliosis had not been confirmed. It outlined the expected clinical symptoms of a patient with Lyme Borreliosis, summarised the diagnostic difficulties in inherent in laboratory diagnosis, particularly the potential for false positive results in low or zero prevalence such as Australia. The position statement also made several recommendations for laboratory investigation of suspected Lyme Borreliosis cases in Australian patients [59]. The Lyme Disease Association of Australia put out its own position statement which was critical of the RCPA's, however it is interesting to note that they too are now labelling this disease as Lyme-like [156].

**Fig. 2:**
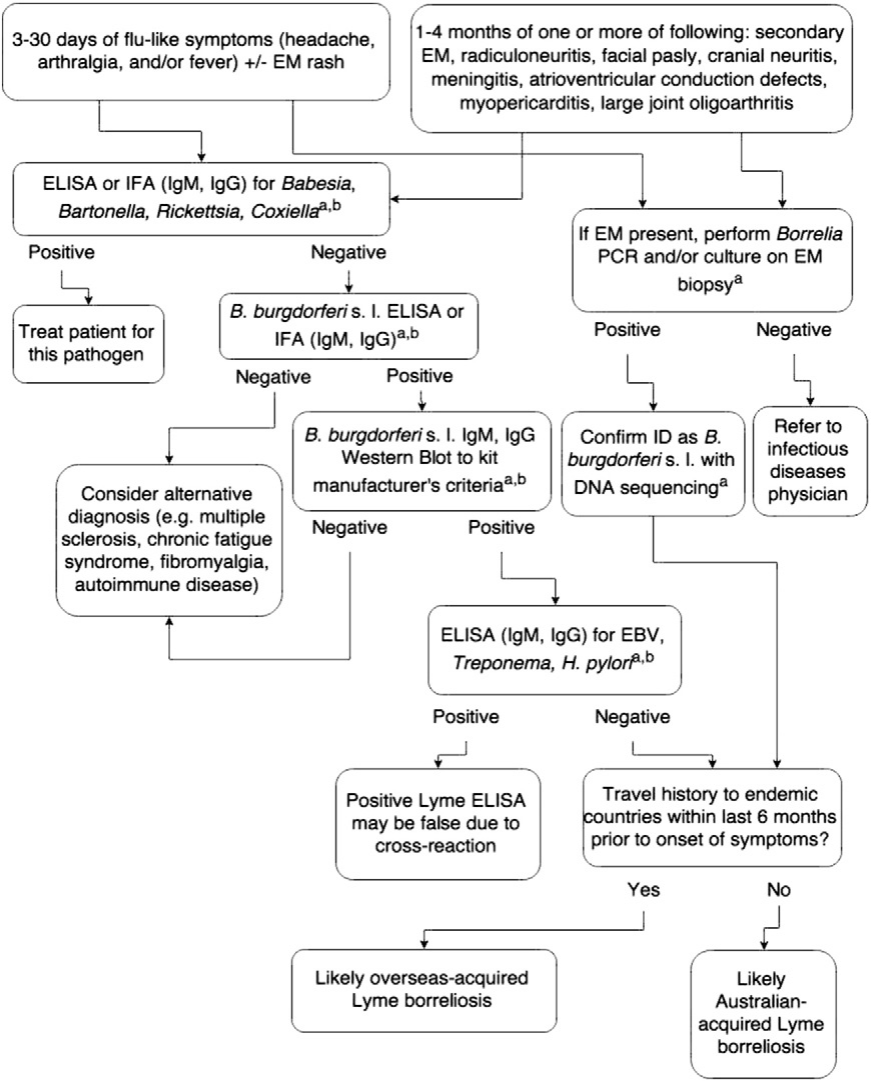
Assessing the cause of a patient's Lyme-like disease. ^a^ Perform only in NATA-accredited laboratory. ^b^ Paired serum testing must be performed. Only consider positive if there is a 4-fold rise in titre, or seroconversion is observed. Positive results without 4-fold rise or seroconversion only indicate past exposure and not current infection. A third serum sample may be required if equivocal. EM, erythema migrans; ELISA, enzyme-linked immunosorbent assay; IFA, immunofluorescence assay; and EBV, Epstein-Barr virus.

It was recommended in the RCPA's position statement that serological diagnosis of Lyme Borreliosis in Australia should consist of an EIA followed by a confirmatory western blot. It is noted that Australian reference laboratories can effectively diagnose Lyme Borreliosis in affected patients who have returned from a known Lyme endemic area who contracted the infection over four weeks previously. Laboratory tests with unconfirmed efficacy of diagnosis, such as measurement of CD57 lymphocyte counts and PCR on urine for the detection of *B. burgdorferi* s. l. DNA, were not indicated as relevant to the diagnosis of Lyme Borreliosis. Importantly, the report recommended that testing should only be performed in NATA/RCPA accredited laboratories, and patients and their doctors were advised to exercise caution in the interpretation of result from non-accredited laboratories in Australia and overseas that have not been validated to diagnose Lyme Borreliosis based upon international consensus documents. The position statement did allow for the culture and PCR of erythema migrans-type rash biopsies collected by interested doctors from patients with no travel history outside Australia for research purposes [59]. The authors of this review encourage such testing (see Fig. 2), as it will allow the collection of data, specimens and (potentially) cultures that may assist in the elucidation of the cause of the Lyme-like illness reported in Australia. If no infectious agents were recovered, over time and with sufficient specimen numbers, a large body of negative evidence by molecular and phenotypic methods from such testing would almost definitively exclude *B. burgdorferi* s. l. as the cause of this illness in Australia.

## 4 Lyme-like case reported in Australia

A literature search for Australian Lyme-like cases was performed using the Google scholar search bar at https://scholar.google.com.au/ and the PubMed Advanced Search Builder at http://www.ncbi.nlm.nih.gov/pubmed/advanced. A boolean search involving “Lyme”, “Disease” and “Australia” was used. The search was limited to Academic Journals only and no time frame was set for the search. A further boolean search using the same limiters was then performed using the terms “*Borrelia*” and “Australia”. Finally, any further Australian-relevant articles referenced within these articles that did not come up in the initial search were obtained. Papers that had Australian authors but were otherwise irrelevant to Australia were removed. At least 525 human cases [79], [80], [81], [82], [84], [85], [86], [87], [88], [89] and two bovine cases [28] of Lyme-like illness have been mentioned in the scientific literature. Only the Lyme-like cases with specified locations are portrayed in Fig. 1, and only those with detailed case presentation, diagnosis and location are presented in Table 1. It should be noted that the majority of these are Lyme*-like* cases that are suspected, but *not confirmed* to represent cases of Lyme Borreliosis. Unreliability of the published case reports in their diagnostic methods means the evidence for Australian Lyme-like cases remains quite unsubstantial and unconvincing.

**Table 1:** Geographic distribution of Australian Lyme-like cases from peer-reviewed scientific literature.

Location	Travel history	Symptoms	Laboratory findings	Diagnosis by culture/PCR^a^	Reference
Lower Hunter Valley, NSW	ND	Insect bite followed by EM with secondary lesions, relapsing arthritis with swelling and pain in the knee and left hip, behavioural change, headaches, memory loss, urinary retention, tachycardia.	Biopsy showed perivascular lymphocytic infiltrate. Synovial fluid from left knee 50 × 10 [6]/L, 70% lymphocytes. CSF raised protein (1.24 g/L) normal cell count. EEG and CAT scan unremarkable. Diagnosed with mild meningoencephalitis. ECG-documented supraventricular tachycardia without evidence of atrioventricular block. Negative arbovirus serology (RRV, Sindbis virus, Australian encephalitis virus)	NP/NP	[82]
Guerilla Bay near Moruya, NSW	ND	Insect bite followed by EM. Weeks after treatment, EM recurred.	Biopsy showed superficial and deep perivascular infiltrate of lymphocytes.	NP/NP	[81]
North Bendalong (between Nowra and Ulladulla), NSW	ND	One month EM, lassitude, polyarthralgia, headaches.	Biopsy showed dense perivascular infiltrate of lymphocytes in full thickness of the dermis, some with eosinophils.	NP/NP	[81]
Gorokan, NSW	ND	3 weeks of increasing lethargy, malaise, intermittent fevers, multiple EM, severe occipital headache, sore throat.	Biopsy showed mixed acute and chronic infiltration in superficial dermis. No spirochaetes on silver staining. ESR and C1q binding assay elevated. Negative culture. Syphilis serology, antinuclear factor, antistreptolysin O titre and immunoglobin all normal levels Paired sera CDC ELISA showed optical density ratio of 0.02 (acute serum) and 0.05 (convalescent serum) when compared with strongly positive reference serum. This is below the 0.2 ratio expected in patients with late or complicated Lyme disease (but this patient only early Lyme disease).	Negative/NP	[85]
Pittwater Shire, Sydney	17 months prior to tick bite, visited 3 countries in Europe known to be endemic for Lyme. Did not recall any tick bites or exposure to ticks. EM appeared at the Australian tick bite site.	EM at tick bite site. Mild headache, malaise and low grade fever, non-pruritic rash, insomnia, generalised arthralgias, myalgias, insomnia, difficulty with memory and "thinking clearly”, secondary EM lesions. Duration > 18 months	ELISA *B. burgdorferi* s. s. antibody negative. Western blot 2 + antibodies (one level below strongest staining intensity) to outer surface protein A (OspA) of *B. garinii* only. Biopsy of secondary lesion showed mild, mainly perivascular lymphohistiocytic inflammatory cell infiltrate in superficial dermis, minimal exocytosis, a little pigment incontinence, no organisms demonstrated with PAS, Gram or Warthin–Starry stains. Culture of biopsy grew spirochaetes Direct immunofluorescence antibody staining to flagellin protein of *B. burgdorferi* s. l. and PCR of the flagellin and 16S rRNA identified *B. garinii* more closely related to European rather than Asiatic *B. garinii* strains.	Positive/positive (NS)	[84]
152.8E, 31.66S	Yes	EM, no systemic illness	*rpoC* PCR positive	NP/positive-sequencing showed 99% identity match to *B. burgdorferi* strain N40	[87]
152.7E 31.73S	Never left Australia	EM, systemic illness	*rpoC* PCR positive	NP/positive-sequencing showed 99% identity match to *B. burgdorferi* strain N40	[87]
151.3E, 33.74S	Yes	EM, fever, meningism, severe headache worse with coughing and shaking of head, photophobia and retro-orbital pain.	*rpoC* PCR positive Follow-up testing post-treatment revealed: *B. burgdorferi* IgA, G, M negative. *B. burgdorferi* multiplex PCR negative (primer targets not specified). *Babesia* and *Bartonella* serology negative.	NP/positive-sequencing showed 98% identity match to *B. burgdorferi* strain N40	[87]
152.8E, 31.32S	Never left Australia	EM, no systemic illness	Multiplex primer set *16S rRNA* and *OspC* used — but only one product sequence is provided. Unclear if both or only one set was positive.	NP/positive-sequencing result inconclusive.	[87]
Rural Victoria	ND	Fever, regular presumed viral illness, chronic fatigue syndrome. Severe arthritis in hands, auditory hypercusis, poor concentration, irritability and emotional lability, episodic sleep disturbances, two episodes of severe generalized body pain without cause, one episode of auditory hallucinations and paranoid ideas. Duration: 8 years	Diagnosed with fibromyalgia at 17 yrs ld Lyme serology IgG titre 80 and IgM titre 10.	NP/NP	[79]
Mid-north coast of NSW	Travelled from Byron Bay NSW to Eastlakes Victoria. No overseas travel.	Lyme-like presentation	Lyme IgM western blot bands 23–25, 39 and 41 kDa. *B. henselae* IgM serology positive (titre 1:40).	NP/NP	[80]
QLD	Travelled to northern NSW and Sydney, NSW; Melbourne, Victoria; Hobart, Tasmania. No overseas travel.	Lyme-like presentation	Lyme IFA 1:40. Lyme IgM western blot bands 31 and 41 kDa. Positive *Borrelia* plasmid PCR. *Babesia duncani* IgG serology positive 1:40, *Babesia microti* IgG serology positive 1:40, *Bartonella henselae* IgM serology positive 1:40.	NP/positive (NS)	[80]
Armstrong beach, QLD	Karratha, WA. No overseas travel.	Lyme-like presentation	Lyme IFA serology 1:80, Lyme IgM western blot bands 34 and 41 kDa, *Bartonella* IgG serology positive 1:40.	NP/negative	[80]
NSW	Victoria, Queensland, South Australia. No overseas travel.	Lyme-like presentation	Lyme IgM western blot bands 31 and 41 kDa. *Babesia duncani* IgG positive 1:40.	NP/negative	[80]

ND, no data; NP, not performed; NS, not sequenced; EM, erythema migrans; ESR, erythrocyte sedimentation rate; PCR, polymerase chain reaction; EEG, electroencephalogram; CAT, computerized axial tomography; ECG, electrocardiogram; CSF, cerebrospinal fluid; RRV, Ross river virus; NT, northern territory; WA, western Australia; QLD, Queensland; NSW, New South Wales; VIC, Victoria; and TAS, Tasmania.

a Serological confirmation of Lyme Borreliosis in the context of non-endemicity is questionable; diagnosis by culture and molecular identification methods are preferable for confirmation in the Australian setting.

### 4.1 Serology from patients

Several patients have been diagnosed as having likely Lyme Borreliosis in Australia solely upon the basis of positive results by one of several methods. The limitations of Lyme serology in Australian patients are discussed in Section 3.2. Over 200 Australian patients (and one Australian cow) presenting with a Lyme-like disease have tested “positive” for Lyme Borreliosis serology [28], [79], [80], [88], [89]. However, 32 of these were diagnosed by IFA or EIA only [28], [79], [89]. None of these one-tiered tested patients (EIA or IFA only) can be definitively considered to have Lyme Borreliosis without further confirmatory testing. Of the 28 positives described by Mayne [80], 15 were immunoblotted without a supporting IFA result being published, severely hindering the validity of these results. A further nine “positive” IgM results are ruled out because of the lack of diagnostic value of the IgM results when the clinical syndrome has been present for greater than 30 days. The remaining four positives had only four or less of the 10 bands required for positive IgG. A further 19 IFAs, 100 IgM immunoblots and 75 IgG immunoblots have also been reported as positive in Australian patients presenting with a Lyme-like condition but also showing concurrent positive antibody titres in several other infectious disease serology tests [88]. It must be reiterated that in a non-endemic or low-endemicity setting, cross reaction of non-specific antibodies due to the presence of other diseases will often lead to the visualisation of false-positive immunoblot bands. In summary, none of the published Lyme-like illness cases from Australian patients diagnosed by serology alone have met the minimum criteria for serological diagnosis of Lyme Borreliosis as described in Section 3.1.

### 4.2 Culture from patients

Although biopsies of erythema migrans have been taken from numerous Australian patients for histology or PCR [81], [82], [85], [87], there has only been one published report of *Borrelia* culture been successful [84]. The case involved a patient that had sustained a tick bite while walking in bushland of Pittwater Shire, Sydney. This was followed by erythema migrans formation, headache and fever, and later generalised arthalgias and myalgias, insomnia and recurrent skin lesions. Over 18 months after the initial tick bite a biopsy of one of the patient's secondary erythema migrans lesions was cultured in BSK-II media. Spirochaetes were present after three weeks incubation and were identified by direct immunofluorescent staining as *B. garinii*. Although the disease appeared to follow the tick bite contracted in New South Wales, this patient had also travelled to three Lyme-endemic countries in Europe 17 months before the onset of his symptoms [84]. Whilst this published case demonstrates a culture confirmed Lyme Borreliosis-causing *Borrelia* isolate in an Australian patient, Australian acquisition could not be confirmed.

### 4.3 Molecular detection of *B. burgdorferi* s. l. from patients

*Borrelia burgdorferi* s. l. DNA has been detected and sequenced in five Australian patients presenting with Lyme-like disease. Three patient erythema migrans biopsies were tested for *B. burgdorferi* s. l. using primers coding for Borrelial *rpoC* [87]. The publication stated that sequencing of the products revealed a 99% homology with *B. burgdorferi* s. s. One of these patients had never left Australia. However, the primer sequences were not published and the three sequences differed significantly in size, being 206 bp, 336 bp and 165 bp long [87], suggesting non-specific cross-priming. Another erythema migrans rash biopsy was tested using a duplex PCR targeting borrelial *ospC* and *16SrRNA.* The paper states that the *ospC* PCR yielded an amplicon 83 bp long [87]. However, analysis of the *ospC* primers utilized in the study using the NCBI Primer Designing Tool (http://www.ncbi.nlm.nih.gov/tools/primer-blast/) shows an expected amplicon of 104–113 bp in length. Thus, non-specific amplification may have led to the positive PCR reaction. The same author reported elsewhere an Australian erythema migrans biopsy yielding a product with a *Borrelia 16SrRNA* PCR once again having a 99% homology with *B. burgdorferi* s. s. [86]. The sequence of the amplicon was not provided, and the primer sequences were also withheld [86]. The laboratory concerned has, to date, yet to share their primer sequences, nor any DNA or isolates with other researchers for independent verification. A further 126 positive *Borrelia* PCRs on blood samples and 46 on urine samples have been reported, but no sequencing was performed to confirm the amplicon identities, and the primers were once again not disclosed [88]. Many of the abovementioned patients also had overseas travel histories [88]. Given the controversy surrounding the possibility of Lyme Borreliosis transmission in Australia, unequivocal demonstration of the local acquisition of *B. burgdorferi* s. l. within this country would be best supported by both a cultured isolate (stored for analysis by other laboratories, including a recognised reference laboratory skilled in the identification of such isolates) and positive direct molecular identification from clinical material (confirmed by sequencing) from a patient with absolutely no history of overseas travel.

### 4.4 Seroprevalence in the population

It would be expected that if the putative Lyme-like disease in Australia is caused by *B. burgdorferi* s. l., there would be a high seropositive rate in the Australian population and an even higher seroprevalence in reservoir hosts. However, the seroprevalence rate of *B. burgdorferi* s. l. using IgG ELISA in residents of coastal New South Wales was found to be 2.2% (9/400) and in dogs of this area the prevalence was 2.5% (6/239) [90]. Conversely, in Westchester County, New York (endemic for Lyme), 49.2% of dogs were seropositive, ranging from 6.5% to 85.2% depending on the municipality [91] and in New Jersey by IFA, 34.7% of asymptomatic dogs were seropositive [92]. In the Aland Islands of Finland (also Lyme endemic), 19.7% of residents were positive for *B. burgdorferi* s. l. IgG with ELISA [93].

## 5 Differential diagnoses

### 5.1 Infectious diseases

It is assumed by many that the causative agent of Lyme-like illness in Australia must be tick-borne. As noted in a previous section, almost all Australian Lyme-like illness predominantly present with a condition analagous to chronic Lyme Borreliosis. Indeed, it is unusual that not more acute Lyme Borreliosis cases are identified in humans and animals within Australia if the organism causing this illness was indeed B. burgdorferi s. l. Any putative agent of the Australian Lyme-like disease would be capable of producing a syndrome similar to Lyme Borreliosis, with a clinical presentation including flu-like symptoms followed by arthralgic, neurological, dermatological and/or cardiac complications. Some Australian bacteria, parasites and viruses individually, or in co-infection with other pathogens, might produce such a syndrome. A summary of known Australian endemic infectious agents that might be considered in the differential diagnosis of an Australian patient with a Lyme-like presentation is presented below.

The clinical presentations of the Australian Rickettsioses are quite similar to each other and atypical presentations may mimic an acute Lyme Borreliosis. Symptoms include headache, chills, malaise, fever, lymphadenopathy, maculopapular rash and an eschar found at the tick bite site [94], [95]. Sometimes arthralgias and myalgias may also be present [94], [96]. In some cases, the eschars may be absent [96], [97] and the rash may appear as varicelliform [94] or petechial [97]. In rare cases, the rash will not develop at all [97]. Rickettsial infections presenting without a maculopapular rash could be mistaken for a Lyme-like illness.

In Australia, *Babesia canis vogeli* is found throughout northern and central Australia and is spread by *Rhipicephalus sanguineus* ticks [98]. *Babesia gibsoni* has been described in dogs in Victoria [98]. *Babesia bovis* and *Babesia bigemina* in cattle are spread by the Australian cattle ticks *R. australis* [99], [100]. *Babesia equi* (later known as *Theileria equi* [101]) was briefly introduced to Australia in 1976 [102], [103] but this did not spread and become established due to the absence of suitable vectors [106]. *B. bovis* has been reported as a rare cause of infection in humans [104]. The first definitive case of human Babesiosis acquired in Australia was reported in 2012 and was caused by *Babesia microti* [105]. To date, *B. microti* has not been identified in any Australian ticks. *Babesia* infection can be atypically associated with rheumatoid muscular pains, and nervous complications including incoordination of legs and hysteria, restlessness and nervousness [106]. It therefore appears that *Babesia* is capable of mimicking a Lyme-like syndrome. Like *B. burgdorferi* s. l., *Babesia* is also capable of establishing long-term, persistent infection [107].

*Coxiella burnetii* may also be considered in patients with tick bite history and reporting Lyme-like symptoms. The majority of cases of *C. burnetii* infection are asymptomatic, but in symptomatic infections the most prevalent acute symptoms include fever (95%), headaches (53%) and myalgia (38%) [108]. Other manifestations may include hepatitis, pneumonia, meningitis, meningoencephalitis, pericarditis and myocarditis [108], [109], [110]. Chronic infection may manifest as endocarditis, vascular infections, osteoarticular infections, chronic hepatitis, pericarditis and very rarely as adenopathies, lung or splenic pseudotumours, or chronic neuropathy [108], [111], [112], [113], [114]. Therefore Q fever may sometimes present as an infection similar to Lyme carditis or Lyme neuroBorreliosis.

Many tick species have been shown as capable of carrying *Bartonella* spp. including: *I. ricinus*, *Dermacentor occidentalis*, *Dermacentor variabilis*, *Dermacentorreticulatus*, *H. longicornis*, *Harperocallis flava*, *Ixodes nipponensis*, *Ixodes pacificus*, *Ixodes persulcatus*, *I. ricinus*, *Ixodes scapularis*, *Ixodes turdus*, *Ixodes antechini*, *Ixodes australiensis*, *I. tasmani*, *Ixodes trichosuri* and *Rhipicephalus sanguineus* [115], [116], [117], [118], [119]. Presently, only *Bartonella henselae* [120], [121], [122], [123], [124] and *Bartonella quintana* [125] have been reported to cause disease in Australian residents. However, a number of other *Bartonella* species of unknown clinical significance have been identified in Australian animals and their parasites [116], [117], [119], [125], [126].

*B. henselae* infection (cat scratch disease) is typically associated with isolated lymphadenopathy with fever without any other symptoms [128]. However it is now recognised that *Bartonella* may cause a wide spectrum of atypical manifestations even in immunocompetent patients [127], [128], [129], [130]. Atypical manifestations may mimic a Lyme-like illness [131] including rheumatic manifestations [131], [132], [133], fibromyalgia and chronic fatigue syndrome [131], [134], neurological disease [135], [136], [137] and endocarditis [138], [139]. *B. henselae* been associated with erythema marginatum rashes [130] that may be mistaken for an erythema migrans rash. Like *B. burgdorferi* s. l., *B. henselae* is capable of sustaining chronic infection [134], [140], [141].

DNA sequences of a newly discovered organism, *Candidatus Neoehrlichia*, were recovered from fifteen New South Wales *I. holocyclus* ticks tested by Gofton, et al. [75]. These sequences did not conform to the emerging tick-borne pathogen *Ca.s Neoehrlichia mikurensis*, but did cluster within two clusters belonging to the *Ca. Neoehrlichia* group [75] and later designated “*Ca. Neoehrlichia* species A and B” [76]. The two species were detected in 248 *I. holocyclus* ticks from both eastern and Western Australia by 16s rRNA next generation sequencing, though when a *Ca. Neoehrlichia* species A and B specific nested PCR was applied to the same samples, only 36 were positive [76]. *Candidatus Neoehrlichia mikurensis* has previously been detected in rodents, humans and ticks from Europe and Asia [75]. A review of eleven human cases in Europe showed that all but one patient were actively immunosuppressed, and most were asplenic [142]. Symptoms included fever, myalgia, arthralgia, neutrophilia and anaemia combined with vascular events such as transient ischaemic attacks and deep vein thrombosis [142]. Only five of the patients recalled being bitten by a tick [142]. While some of these described symptoms may be confused with a Lyme-like illness, further work must be performed to determine the host range, infectivity and clinical presentation of the two novel *Ca. Neoehrlichia* species detected in Australian *I. holocyclus* ticks before these may be confirmed as potential Lyme-like disease candidates. Furthermore, other novel candidate infectious agents such as the three new species each of *Anaplasma* and *Ehrlichia* that have been identified by next generation sequencing of Australian ticks, though at much lower prevalence than the novel species of *Ca. Neoehrlichia* species, also require investigation [76].

### 5.2 Non-infectious diseases

It is important that potential non-infectious causes are considered in the investigation of Australian patients presenting with a Lyme-like illness. Fibromyalgia, chronic fatigue syndrome, delusional parasitosis and multiple sclerosis are examples of conditions that may be misdiagnosed as a Lyme-like disease, especially in Australia where the infectious aetiology for this condition has not been elucidated. This list is by no means exhaustive.

It should be noted that antigens in *I. holocyclus* saliva alone may cause an erythematous rash to develop in bitten patients [143]. Of forty-two volunteers inoculated by pin-prick with an extract of *I. holocyclus* salivary glands, 36% developed a local erythematous lesion at that site within minutes or hours [143]. In most cases, the rash was > 50 mm in diameter and persisted for up to 7 days or more [143]. Such a hypersensitivity rash might easily be mistaken for an erythema migrans lesion in patients recently bitten by *I. holocyclus* ticks [143]. These findings do raise a question as whether the Australian presentations of a Lyme-like illness may in some cases be an allergic response by some individual patients to antigens found within local tick saliva.

Symptoms of fibromyalgia include widespread musculoskeletal pain, hyperalgesia, fatigue, insomnia, memory loss and poor concentration, depression, headache and irritable bowel syndrome [144], [145], [146]. Since diffuse arthralgia, cognitive difficulties and fatigue are common in chronic Lyme Borreliosis, it is possible for fibromyalgia to be mistaken for Lyme borrelioisis and *vice versa* [147], [148].

Chronic fatigue syndrome is very similar to fibromyalgia in that it is a syndrome of unknown aetiology characterised by persistent fatigue, musculoskeletal pain, insomnia and cognitive impairment and headaches [149], [150], [151]. Both syndromes are more common in women than men, and the two syndromes commonly co-occur. It has even been suggested that the two syndromes are merely symptom amplification of the same somatic syndrome [149]. Fibromyalgia is diagnosed based on widespread musculoskeletal pain, sensitivity in a number of “tender spots”, and the presence of other associated symptoms such as headaches, sleep disturbances and memory loss [152]. Chronic fatigue syndrome diagnosis is based on onset of unexplained persistent or relapsing chronic fatigue that is not substantially alleviated by rest, accompanied by symptoms such as short term memory or poor concentration, sore throat or lymph nodes, muscle or joint pain and headaches [150]. Chronic fatigue and fibromyalgia may present as sequelae of infections with *C. burnetii*, *Chlamydophila pneumoniae*, Epstein-Barr virus and Parvovirus B19 [150].

Delusional parasitosis is a psychiatric disorder where a patient has the false but fixed belief that they are being infested by parasites [153], [154]. It may present as a primary somatic disorder or secondary to other conditions such as drug use, schizophrenia or dementia. Primary delusional parasitosis occurs most commonly in middle-aged women, and except for their delusion the patient may otherwise be rational and mentally healthy [153]. Patients may describe sensations of parasitic activity on or under their skin such as crawling, biting or burrowing (collectively known as formication), and may bring in objects such as hair, lint or skin as evidence of their infestation despite unremarkable findings on examination [153], [154].

There has been one published Australian case of delusional parasitosis in which the patient was convinced she had Lyme Borreliosis [155]. The patient brought evidence of “ticks” to her doctor and presented with rashes as a result of scratching and disinfecting. The patient had shaved off all her hair and fumigated her house in an attempt to be rid of the arthropods. However after several months of cognitive behavioural therapy and 150 mg of venlafaxine, her paranoia and symptoms were successfully alleviated [155].

## 6 Conclusion

Suggestions that a Lyme-like disease may exist in Australia [17] remain controversial and no study to date has definitively identified the presence of a *Borrelia* species infecting humans that have a locally acquired Lyme-like syndrome. It is unclear whether the causative agent of this purported condition is a *B. burgdorferi* s. l. related organism, another pathogen altogether or of non-infectious aetiology. Over 500 Lyme-like cases from Australian patients have been published in the scientific literature [79], [80], [81], [82], [84], [85], [86], [87], [88], [89] and two bovine cases [28] but upon investigation, these diagnoses were highly questionable due to significant flaws in the diagnostic process or presentation of results. Only in one instance has a Lyme Borreliosis-causing *Borrelia* species been cultured from an Australian patient or animal [84]. This patient had a history of travel to a Lyme endemic area of the northern hemisphere [84] so overseas acquisition cannot be ruled out. Serology has a low positive predictive value in non-endemic areas and cannot be relied upon for diagnosis. The reported culture of possible *Borrelia* spirochaetes from 109 Australian ticks [66] was not reproduced in over 10,000 ticks [73]. *B. burgdorferi* s. l. has never been cultured from an Australian patient that could not have acquired the infection overseas and therefore there is currently no proof that *B. burgdorferi* s. l. or any other kinds of *Borrelia* species are infecting humans in Australia. If there is a Lyme-like disease that exists in Australia it may well be of a different aetiology. It is recommended by the authors that in the non-endemic context such as Australia, in addition to following the RCPA protocol for the diagnostic laboratory testing of Borreliosis [59], a minimum of live *Borrelia* culture combined with a positive, sequenced *B. burgdorferi* s. l. specific PCR and independent verification of the identity of that organism by an experienced reference laboratory is required to confirm any future diagnosis of Australian acquired Lyme Borreliosis.

## Disclaimer

Richard Bradbury is co-authoring this article in his personal capacity and in his capacity as an adjunct academic at Central Queensland University.
